# Functional and oncologic outcomes of robot-assisted simple enucleation with and without renal arterial cold perfusion in complex renal tumors: a propensity score-matched analysis

**DOI:** 10.1186/s12894-020-00771-7

**Published:** 2021-01-06

**Authors:** Qun Lu, Xiaozhi Zhao, Changwei Ji, Suhan Guo, Xuefeng Qiu, Guangxiang Liu, Shiwei Zhang, Xiaogong Li, Gutian Zhang, Xuebin Zhang, Hongqian Guo

**Affiliations:** 1 Department of Urology, Drum Tower Hospital, Medical School of Nanjing University, Institute of Urology, Nanjing University, 321 Zhongshan Rd., Nanjing, 210008 Jiangsu People’s Republic of China; 2 School of Public Health, Nanjing Medical University, Nanjing, 210029 Jiangsu People’s Republic of China; 3 Department of Radiology, Drum Tower Hospital, Medical School of Nanjing University, Nanjing, 210008 Jiangsu People’s Republic of China

**Keywords:** Renal cell carcinoma, Enucleation, Robotic partial nephrectomy, Perfusion, Hypothermia

## Abstract

**Background:**

To compare robot-assisted simple enucleation with renal arterial cold perfusion (RACP-RASE) and RASE alone in complex renal tumors with regard to perioperative, functional and oncologic outcomes by propensity score-matched analysis.

**Methods:**

Data from 351 patients who underwent RACP-RASE or RASE for complex renal tumors were recorded between September 2014 and December 2017. Propensity score-matched analysis was performed on age, sex, BMI, ECOG score, tumor side and size, preoperative estimated glomerular filtration rate (eGFR), RENAL score and PADUA score.

**Results:**

The study included 31 RACP-RASE and 320 RASE procedures. RENAL score and PADUA score were higher and tumor diameter was greater under RACP-RASE than RASE. After matching, the two groups were similar in estimated blood loss (208.3 vs 230.7 ml; *p* = 0.696) and ischemic time (34.8 vs 32.8 min; *p* = 0.342). The RACP-RASE group had significantly longer operative time than the RASE group (264.1 ± 55.7 vs 206.9 ± 64.0 min, *p* = 0.001). There was no difference in the incidence of postoperative complications between the two groups (13.8% vs 24.1%; *p* = 0.315), as was the overall incidence of positive surgical margins (3.4 vs 0%; *p* = 1.000). The changes in eGFR significantly differed between the two groups at 3 months (*p* = 0.018) and 12 months (*p* = 0.038). More patients in the RASE group were CKD upstaged (*p* = 0.043). At multivariable analysis, preoperative eGFR and the type of procedure were significant predictive factors for a change of more than 10% in eGFR at 3 months postoperatively. There was no local recurrence or distant metastasis during follow-up.

**Conclusions:**

RACP-RASE is an effective and safe technique for complex renal tumors that can provide appropriate temporary arterial occlusion and renal hypothermic perfusion. Renal arterial cold perfusion may be helpful in protecting renal function in RASE as compared with warm ischemia.

## Background

An increasing number of patients with renal tumors have been detected at an early stage because of the widespread practice of health examination. Partial nephrectomy (PN) has become the standard therapy for treating localized renal tumors, and it provides desirable oncological and functional results not inferior to radical nephrectomy [1]. Traditionally, standard partial nephrectomy (SPN) includes removal of the tumor and a visible rim of healthy parenchyma [2, 3]. Simple enucleation (SE) is an alternative nephron-sparing technique that minimizes the thickness of normal parenchyma surrounding the tumor [4, 5]. About 82% of renal masses have a demonstrable pseudocapsule [6], providing an avascular plane to enucleate the mass. Several studies have revealed that SE can preserve more normal renal parenchyma than SPN [4, 7].

Renal ischemia during PN may result in postoperative kidney insufficiency due to ischemia/reperfusion injury. Although it is generally considered that temporary hilar control during PN won’t affect the short-term renal function significantly [8, 9], decline in renal function averages about 20% in the operated kidney [10]. The risk factors like advanced age, hypertension, or the ischemia time exceeding 30 min further aggravate kidney injury. Some studies have reported that organ hypothermia has been applied to prolong the ischemia time and decrease the degree of kidney damage [11]. Lowering the renal temperature can decrease the renal metabolism and limit the hypoxia-induced injury pathways [12]. Several ways of hypothermic protection have been reported, such as surface cooling with ice slush, renal arterial cold perfusion using angiography, and retrograde transureteral perfusion.

With advanced characteristics such as magnified 3-D vision with high definition quality and precise control with articulating instruments, the da Vinci surgical system may overcome the disadvantages present in laparoscopic PN and significantly shorten the learning curve [13]. The surgical indications for robotic PN have expanded to more complex renal lesions, like completely endophytic or hilar tumors.

In this study, we present our initial experience with renal function protection technology combining robot-assisted simple enucleation (RASE) and renal arterial cold perfusion (RACP-RASE) for treating complex renal tumors. We describe the technique detailedly and present the results on perioperative, functional and oncologic outcomes comparing RACP-RASE and RASE alone.

## Methods

### Patients

The data of 351 patients who underwent RASE or RACP-RASE for clinical localized renal tumors were collected from September 2014 to December 2017. We detected the perioperative, functional and oncologic date. All the procedures were completed by one surgeon (Hongqian Guo). According to the tumor characteristics and our experience, we assessed the ischemia time during SE preoperatively. If the ischemia time may well exceed 30 min, we would perform RACP-RASE. The patients in RACP-RASE group were mostly with complex renal tumors, including endophytic, hilar, and renal tumors larger than 7 cm, which accounted for 77.4% of patients. The rest had a deep single lesion with renal sinus or collecting system contact in which prolonged ischemia time was anticipated. All patients in RACP-RASE group received a preoperative consultation of the Department of Radiology Intervention, and the interventional radiologist developed a proposal according to the three-dimensional reconstruction of renal artery. The study was approved by the local ethics committee, and all patients signed the informed consent.

Demographic data and perioperative outcomes of all patients were obtained. Level of functioning was classified by the Eastern Cooperative Oncology Group (ECOG) criteria [14]. The tumor presentation pattern was classified according to the Patard classification [15]. The score of renal tumors were determined by an experienced radiologist in conjunction with an urologist on the basis of the RENAL nephrometry score system and PADUA nephrometric classification [16, 17]. Postoperative complications were stratified according to the Clavien–Dindo grading system [18]. Estimated glomerular filtration rate (eGFR) calculated by the modification of diet renal disease formula was used to assess the kidney function [19]. Chronic kidney disease (CKD) was defined by the National Kidney Foundation Kidney Disease Outcome Quality Initiative classification. We counted the time of balloon catheterization into total operative time in RACP-RASE group.

### Surgical technique

Before anesthesia the patient was first sent to the Radiology Intervention Department, and the renal artery balloon catheter was punctured through the femoral artery to achieve cold perfusion under the guidance of fluoroscopy. The interventional radiologist inserted a 5.5Fr balloon angiocatheter in the renal artery as distally as possible to avoid accidental shifting of the catheter. If the trunk of the renal artery was short, a 4Fr balloon catheter was placed in each of the two branches of the renal artery. The patient was then taken to the operative room in a horizontal position. After anesthesia the patient was placed in the lateral position. The Si da Vinci surgical system (Intuitive Surgical, Sunnyvale, CA, USA) was used for RASE. We employed two 8-mm ports for the instruments and one 12-mm port for the camera, and the transperitoneal approach was routinely used. The Fenestrated or Maryland grasper on the left arm and the monopolar scissor on the right arm were generally used in RASE.

**Fig. 1:**
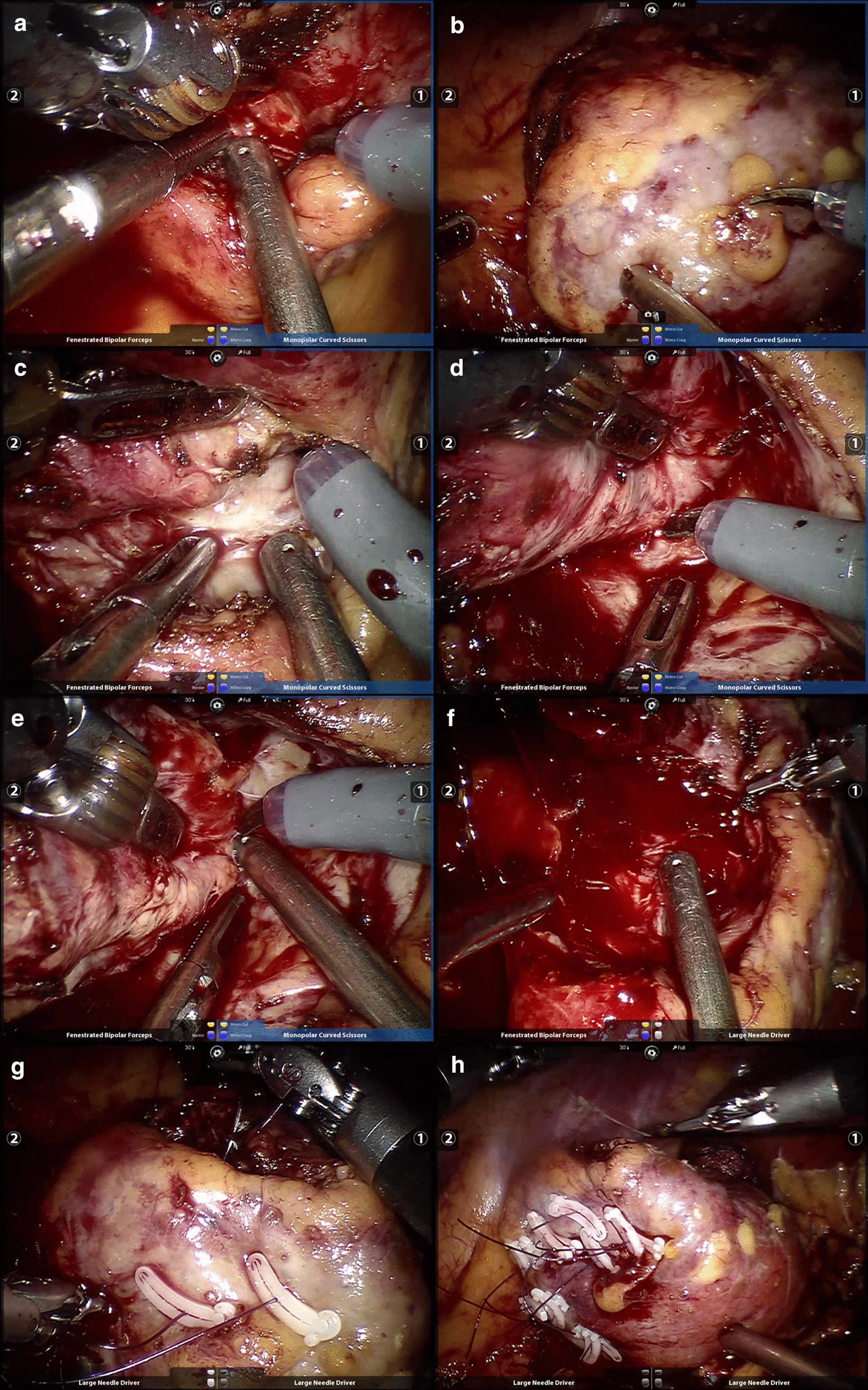
Intraoperative showing: incision of the renal parenchyma near the tumor edge until the tumor pseudocapsule is identified or the bleeding is interfering with the safe removal of the tumor (**A**). The renal artery is blocked by an inflating balloon, and cold ischemia is achieved by perfusion of chilled Ringers until the renal parenchyma becomes bleached (**B**). Removing the tumor with blunt dissection by using the natural cleavage plane between the tumor and normal parenchyma (**C**–**E**). The renal surface is examined to rule out tumor infiltration and hemorrhage in the tumor bed is controlled by bipolar coagulation without sutures conventionally (**F**). The single-layer renorrhaphy technique is performed for renal reconstruction (**G**). Deflating the balloon and inspecting the parenchymal defect (**H**)

The method of tumor enucleation was described in our previous study [20]. The enucleation began with a cold incision a few millimeters around the lesion without ischemia. When the tumor capsule was seen or hemorrhage blurred the surgical field, the renal artery was blocked by inflating 0.5–1.0 ml saline solution into the catheter balloon. Cold ischemia was achieved by perfusion of chilled Ringers lactate through the catheter at the rate of 20 ml/min, and the enucleation wound contributed to the outflow of the perfusion fluid. The surgeon then enucleated the tumor without visible normal parenchyma around it by blunt dissection. After the tumor was removed, the tumor bed was easily detected to rule out possible tumor infiltration because the renal parenchyma became bleached. Hemostasis was controlled by bipolar coagulation, and the collecting system fracture was ligated using a single suture. The cortical defect was usually closed with horizontal and interrupted sutures. Then the perfusion was stopped and the balloon was deflated. Finally, the parenchyma defect was detected to rule out bleeding, and additional sutures was employed if necessary (Fig. 1).

### Pathology

Tumors pathology were staged according to the TNM classification [21]. The histological subtypes of tumor were classified by the 2004 version of WHO classification. The nuclear grade was classified by the 2016 WHO/ISUP grading [22]. All specimens were grossly analyzed for surgical margin evaluation.

### Statistical analysis

We used the Mann–Whitney U-test or the Pearson chi-square test to compare the variables. A propensity score-matched analysis was used to minimize the selection bias of treatment. The propensity score was derived from a multivariable logistic model including the variables of age, sex, BMI, ECOG score, tumor side and size, preoperative eGFR, RENAL score and PADUA score. According to the propensity score, the RACP-RASE patients were matched 1:1 without replacement to the RASE patients by using nearest-neighbour matching within a caliper set at 0.05. A 2-sided *p* value < 0.05 was considered statistically significant and the analysis was performed using SPSS 17.0 (SPSS Inc., Chicago, IL).

## Results

The characteristics of the patients before and after the propensity score matching were shown in Table 1. This study included 31 patients who underwent RACP-RASE and 320 who underwent RASE. The two groups were comparative in age, sex, BMI, ECOG score, symptoms and preoperative eGFR. Anatomical complexity scores (RENAL score and PADUA score) were higher and tumor diameter was greater for patients with RACP-RASE than RASE.

**Table 1:** Preoperative patient data and tumor characteristics before and after propensity-score matching

	Pre-matching			Post-matching		
	RACP-RASE, n = 31	RASE, n = 320	*p* value	RACP-RASE, n = 29	RASE, n = 29	*p* value
Age, years, mean ± SD	52.9 ± 12.9	53.7 ± 13.6	0.695	52.5 ± 13.2	54.4 ± 14.3	0.460
Sex (n, %)			0.810			0.780
Male	19 (61.3%)	189 (59.1%)		19 (65.5%)	20 (69.0%)	
Female	12 (38.7%)	131 (40.9%)		10 (34.5%)	9 (31.0%)	
BMI, mean ± SD	24.6 ± 3.4	24.2 ± 3.1	0.714	24.3 ± 3.1	24.1 ± 3.2	0.646
ECOG (n, %)			0.547			0.368
0	21 (67.7%)	233 (72.8%)		20 (69.0%)	23 (79.3%)	
≥ 1	10 (32.3%)	87 (27.2%)		9 (31.0%)	6 (20.7%)	
Side (n, %)			0.645			0.421
Left	18 (58.1%)	172 (53.8%)		16 (55.2%)	19 (65.5%)	
Right	13 (41.9%)	148 (46.2%)		13 (44.8%)	10 (34.5%)	
Symptoms at diagnosis (n, %)			0.706			0.389
Asymptomatic	27 (87.1%)	291 (90.9%)		25 (86.2%)	27 (93.1%)	
Symptomatic	4 (12.9%)	29 (9.1%)		4 (13.8%)	2 (6.9%)	
Preoperative eGFR, ml/min/1.73m^2^, median (IQR)	101 (80–111)	97 (80–116)	0.960	98 (79–109)	91 (76–110)	0.539
Clinical tumor size, cm, mean ± SD	5.1 ± 1.3	3.9 ± 1.6	**0.000**	5.0 ± 1.7	4.9 ± 1.8	0.870
RENAL score, median (IQR)	10 (10–11)	8 (6–9)	**0.000**	10 (10–11)	10 (10–11)	0.709
PADUA score, median (IQR)	11 (11–12)	9 (7–10)	**0.000**	11 (11–12)	11 (11–12)	0.683

Bold indicated that the *p* value was statistically significant

*RACP-RASE* robot-assisted simple enucleation with renal arterial cold perfusion, *RASE* robot-assisted simple enucleation, *BMI* body mass index, *ECOG* Eastern Cooperative Oncology Group, *eGFR* estimated glomerular filtration rate, *IQR* interquartile range

Within the propensity score matched cohort, a total of 58 patients were matched, with 29 patients in each group. Complex tumor features after matching were shown in Table 2. In the RACP-RASE group, 5 patients had tumors larger than 7 cm, 6 patients had completely intrarenal tumors, 12 patients had hilar tumors, and 1 patient had multiple tumors. The rest had a deep single lesion with renal sinus or collecting system contact. The RACP-RASE group had more hilar tumors than RASE group (41.4 vs 13.8%, *p* = 0.019).

**Table 2:** Complex tumor features after propensity-score matching

	Post-matching		
	RACP-RASE, n = 29	RASE, n = 29	*p* value
Larger than 7 cm	5 (17.2%)	3 (10.3%)	0.703
Completely intrarenal tumor	6 (20.7%)	4 (13.8%)	0.487
Hilar tumor	12 (41.4%)	4 (13.8%)	**0.019**
Multiple tumors	1 (3.4%)	0 (0.0%)	1.000

Bold indicated that the *p* value was statistically significant

Surgical, pathological and functional results before and after matching were shown in Table 3. After matching, the RACP-RASE and RASE groups were similar in ischemic time (34.8 ± 9.4 vs 32.8 ± 7.2 min) and estimated blood loss (208.3 ± 93.8 vs 230.7 ± 135.7 ml). The operative time of RACP-RASE group was significantly longer than the RASE group (264.1 ± 55.7 vs 206.9 ± 64.0 min, *p* = 0.001). The rate of renal sinus entry was comparable (58.6% vs 48.3%). The RACP-RASE group had 1 conversion (3.4%) to radical nephrectomy for oncological reasons, and the RASE group had 1 conversion (3.4%) to radical nephrectomy for significant hemorrhage. The 2 groups did not differ in the incidence of postoperative complications (13.8% vs 24.1%, *p* = 0.315). The complications were mainly Clavien–Dindo grade 1 or 2 complications. Clavien–Dindo grade 3 complications occurred in no RACP-RASE patients and in 1 RASE patient. Overall, 89.7% of RACP-RASE patients and 82.8% RASE patients had malignant tumors, and clear cell carcinoma was the most common histotype. There was no difference in the rate of positive surgical margins between 2 groups (3.4 vs 0%, *p* = 1.000). The only 1 patient with positive margin in RASE group showed no local recurrence to date.

**Table 3:** Surgical, pathological and functional outcomes for RACP-RASE and RASE before and after propensity-score matching

	Pre-matching			Post-matching		
	RACP-RASE, n = 31	RASE, n = 320	*p* Value	RACP-RASE, n = 29	RASE, n = 29	*p* Value
Operative time, min, mean ± SD	262.6 ± 57.1	175.6 ± 45.5	**0.000**	264.1 ± 55.7	206.9 ± 64.0	**0.001**
Ischemic time, min, mean ± SD	35.9 ± 10.8	22.8 ± 7.3	**0.000**	34.8 ± 9.4	32.8 ± 7.2	0.342
Estimated blood loss, ml, mean ± SD	212.6 ± 94.2	179.1 ± 123.6	**0.005**	208.3 ± 93.8	230.7 ± 135.7	0.696
Entry into sinus (n, %)	19 (61.3%)	63 (19.7%)	**0.000**	17 (58.6%)	14 (48.3%)	0.430
Conversions (n, %)						
Open conversion	0 (0.0%)	2 (0.6%)	1.000	0 (0.0%)	0 (0.0%)	–
Radical conversion	1 (3.2%)	6 (1.9%)	1.000	1 (3.4%)	1 (3.4%)	1.000
Length of stay, d, mean ± SD	7.9 ± 1.4	7.9 ± 2.0	0.429	7.8 ± 1.4	8.6 ± 2.3	0.370
Postoperative complications (n, %)	4 (12.9%)	35 (10.9%)	0.973	4 (13.8%)	7 (24.1%)	0.315
Clavien 1–2	4 (12.9%)	31 (9.7%)		4 (13.8%)	6 (20.7%)	
Clavien 3–4	0 (0.0%)	4 (1.3%)		0 (0.0%)	1 (3.4%)	
Tumor histology (n, %)			1.000			0.703
Malignant	28 (90.3%)	286 (89.4%)		26 (89.7%)	24 (82.8%)	
Benign	3 (9.7%)	34 (10.6%)		3 (10.3%)	5 (17.2%)	
Positive margins (n, %)	1 (3.2%)	5 (1.6%)	1.000	1 (3.4%)	0 (0.0%)	1.000
Postoperative eGFR, ml/min/1.73m^2^, median (IQR)						
3 months	93 (73–108)	92 (79–110)	0.670	93 (74–107)	84 (60–105)	0.287
12 months	88 (73–114)	93 (81–112)	0.453	88 (77–108)	80 (61–98)	0.186
Acute renal injury (n, %)	1 (3.2%)	3 (0.9%)	0.795	0 (0.0%)	1 (3.4%)	1.000
Postoperative change in eGFR, %, median (IQR)						
3 months	− 6.3 (− 11.3 to − 2.4)	− 3.2 (− 19.4 to − 2.3)	0.849	− 6.3 (− 10.3 to − 2.4)	− 12.0 (− 17.5 to − 6.7)	**0.018**
12 months	− 5.8 (− 15.5 to 1.9)	− 2.0 (− 17.4 to 10.0)	0.551	− 5.8 (− 12.7 to 0.1)	− 11.7 (− 18.9 to − 4.0)	**0.038**
CKD upstaging n (%)	6 (19.4%)	68 (21.3%)	0.805	5 (17.2%)	12 (41.4%)	**0.043**

Bold indicated that the *p* value was statistically significant

Regarding the renal functional outcomes after matching, the median eGFR 3 months postoperatively was 93 and 84 ml/min per 1.73 m^2^ (*p* = 0.287) for the RACP-RASE and RASE groups, with a median -6.3 and -12.0 percentage change, respectively (*p* = 0.018). Only 1 patient in the RASE group had acute renal injury. The median eGFR 12 months postoperatively was 88 and 80 ml/min per 1.73 m^2^, respectively (*p* = 0.186), with a median − 5.8 and − 11.7 percentage change (*p* = 0.038). The changes in eGFR significantly differed between the two groups. A total of 17.2% of patients in the RACP-RASE group and 41.4% in the RASE group were CKD upstaged (*p* = 0.043). No patient required temporary or permanent dialysis. At multivariable analysis, preoperative eGFR and the type of procedure were significant predictive factors for a change of more than 10% in eGFR at 3 months postoperatively (Table 4).

**Table 4:** Multivariate analysis for a change of more than 10% in eGFR at 3 months postoperatively

Variable	OR	CI	*p* value
Age	0.955	0.894–1.019	0.163
ECOG	1.308	0.305–5.602	0.717
Clinical tumor size	1.196	0.684–2.093	0.530
PADUA score	1.952	0.668–5.707	0.222
Preoperative eGFR	0.968	0.941–0.997	**0.029**
RASE vs RACP-RASE	0.090	0.020–0.408	**0.002**

Bold indicated that the *p* value was statistically significant

The median follow-up with RACP-RASE and RASE was 22 months (range 15–30) and 28 months (range 14–45). No local recurrence or distant metastasis occurred during follow-up.

## Discussion

Here we compared the perioperative, functional and oncologic outcomes of RACP-RASE and RASE alone by propensity score-matched analysis. After matching, the two groups were similar in estimated blood loss and ischemic time. Operative time was longer in RACP-RASE group than RASE group. The main reason was that we counted the time of balloon catheterization into total operative time in RACP-RASE group. It also took minutes to transport patients from the Radiology Intervention Department to the operative room. The total incidence of postoperative complications was comparable between two groups, as was the incidence of positive surgical margins. The changes in eGFR significantly differed between the two groups at 3 months and 12 months. RACP-RASE is a safe and acceptable technique for complex renal tumors that can achieve temporary control of renal artery and provide hypothermic perfusion for the kidney. Renal arterial cold perfusion was helpful in protecting renal function in RASE as compared with warm ischemia.

Nephron-sparing surgeries, including SE and SPN, offer perioperative outcomes similar to those of radical nephrectomy with the advantage of renal function preservation [23, 24]. Minimally invasive surgery especially robotic surgery has the potential benefits for postoperative rapid recovery. In previous studies various techniques of renal cooling in PN have been reported. Gill et al. described an ice slush technique in an open approach with good functional outcomes [25]. Cold ureteral perfusion was also used in laparoscopic PN but without an outcome study [26].

Renal arterial cold perfusion is the most common physiological form of renal hypothermia, and the method has been applied in clinical practice for many years [27, 28]. It replaces the residual blood of kidney and cools the renal parenchyma quickly. Moreover, it prevents intravascular coagulation and makes the surgical visibility clearer. Cold perfusion is also used in kidney transplantation for the organ preservation and in vivo PN through open or laparoscopic approaches [29, 30]. To our knowledge, this is the first study to combine the technology of renal arterial cold perfusion and RASE.

The balloon catheters have the advantage of providing renal hypothermia and renal artery block in RASE for larger tumors or completely intrarenal tumors. The patients should be detailly evaluated, and the catheter should be placed as distally as possible in the renal artery, because of the easy shifting or insufficient occlusion of the balloon. Moreover, any major movement of the body should also be avoided.

Tumor enucleation has been demonstrated in several previous studies. It basically means of enucleate the tumor by blunt dissection along the pseudocapsule as anatomical landmark, without visible normal parenchyma [31]. Therefore the SE technique could maximumly preserve normal renal parenchyma theoretically [5, 32], and it could avoid influencing the renal function by reducing the ischemia time. Moreover, the single-layer renorrhaphy could avoided compressing the deep vessels and reduced the suture time, thereby reducing the ischemic necrosis of the sutured parenchyma.

We observed a median of − 6.3 and − 12.0 percentage change at 3 months, and a median of − 5.8 and − 11.7 percentage change at 12 months in the RACP-RASE and RASE groups, respectively. The changes in eGFR significantly differed between the two groups. More cases in the RASE group were CKD upstaged compared with RACP-RASE group. No postoperative acute renal failure or renal replacement therapy occurred. Multivariable analysis revealed that preoperative eGFR and the procedure type were significant predictive factors for a more than 10% change in eGFR at 3 months postoperatively. A major restriction of RASE technique is time because kidney injury is expected if the ischemia time exceeds 30 min. Lowering the renal temperature can decrease the renal metabolism and limit the hypoxia-induced injury pathways. Therefore, RACP-RASE may be applied to decrease the degree of kidney damage and prolong the ischemia time.

There are several limitations to this study. First, this is a retrospective and small sample study. Second, this study lacks of randomization. Third, this study lacks a standardized manner of the procedure choice which was mainly based on the tumor complexity, and this leads to selection bias.

## Conclusions

Our initial experience with RACP-RASE technique revealed that renal arterial cold perfusion can provide appropriate temporary arterial occlusion and renal hypothermic perfusion during RASE. Renal arterial cold perfusion may be helpful in protecting renal function in RASE as compared with warm ischemia. RACP-RASE is an effective and safe technique with complex tumors such as completely intrarenal and hilar tumors. Long-term study with large patient sample is needed.

## Data Availability

The datasets used and analysed during the current study are available from the corresponding author on reasonable request.

## Abbreviations

PN: Partial nephrectomy
SPN: Standard partial nephrectomy
SE: Simple enucleation
RASE: Robot-assisted simple enucleation
RACP-RASE: Robot-assisted simple enucleation with renal arterial cold perfusion
ECOG: Eastern Cooperative Oncology Group
BMI: Body mass index
